# Does it matter who harmed whom? A cross-cultural study of moral judgments about harm by and to insiders and outsiders

**DOI:** 10.1007/s12144-023-04986-3

**Published:** 2023-07-22

**Authors:** Paul McKee, Hyo-eun Kim, Honghong Tang, Jim A. C. Everett, Vladimir Chituc, Toni Gibea, Lucas Murrins Marques, Paulo Boggio, Walter Sinnott-Armstrong

**Affiliations:** 1 Center for Cognitive Neuroscience, Duke University, Durham, NC USA; 2 Duke Institute for Brain Sciences, Duke University, Durham, NC USA; 3 Department of Humanities, Hanbat National University, Daejeon, Korea; 4 Business School, Beijing Normal University, Beijing, China; 5 School of Psychology, University of Kent, Canterbury, UK; 6 Department of Psychology, Yale University, New Haven, CT USA; 7 Department of Philosophy and Social Sciences, Bucharest University of Economic Studies, Bucharest, Romania; 8 Research Centre for Applied Ethics, University of Bucharest, Bucharest, Romania; 9 Instituto de Medicina Física e Reabilitação, Hospital das Clínicas HCFMUSP, Faculdade de Medicina, Universidade de São Paulo, São Paulo, SP Brasil; 10 Social and Cognitive Neuroscience Laboratory and Developmental Disorders Program, Center for Health and Biological Sciences, Mackenzie Presbyterian University, Sao Paulo, Brazil; 11 Department of Philosophy, Duke University, Durham, NC USA; 12 Kenan Institute for Ethics, Duke University, Durham, NC USA

**Keywords:** Black sheep effect, Moral judgments, Ingroup, Outgroup, Agent, Victim

## Abstract

This cross-cultural study compared judgments of moral wrongness for physical and emotional harm with varying combinations of in-group vs. out-group agents and victims across six countries: the United States of America (N = 937), the United Kingdom (N = 995), Romania (N = 782), Brazil (N = 856), South Korea (N = 1776), and China (N = 1008). Consistent with our hypothesis we found evidence of an insider agent effect, where moral violations committed by outsider agents are generally considered more morally wrong than the same violations done by insider agents. We also found support for an insider victim effect where moral violations that were committed against an insider victim generally were seen as more morally wrong than when the same violations were committed against an outsider, and this effect held across all countries. These findings provide evidence that the insider versus outsider status of agents and victims does affect moral judgments. However, the interactions of these identities with collectivism, psychological closeness, and type of harm (emotional or physical) are more complex than what is suggested by previous literature.

**Supplementary information:**

The online version contains supplementary material available at 10.1007/s12144-023-04986-3.

## Introduction

Most traditional Western philosophical theories (including utilitarianism, deontology, and contractarianism) agree that morality must be impartial (Jollimore, 2021). On these views, whether an act is morally right or wrong—as well as the degree to which it is morally right or wrong—does not depend directly on the identity of either the agent or the victim but only on the kind of act that was done and/or its consequences. Who X and Y happen to be are not supposed to affect how wrong it is for X to harm or lie to Y, for example.

Some philosophers believe that human moral judgments are not as impartial as traditional theories require. Hume (2007) suggested that we sympathize more with people who are closer to us, such as acquaintances. Some ethicists argue that partiality is allowed in some cases, such as parents favoring their own children (Jollimore, 2021).

In line with Hume and his followers, psychologists have found evidence that our moral judgments are affected by whether the agent is seen as an insider (an ingroup member) or outsider (an outgroup member). Different studies, however, point in conflicting directions. Some have found a tendency to judge friends or other ingroup members less harshly than strangers or outgroup members when they commit immoral acts (Valdesolo & DeSteno, 2007; De Bock et al., 2013; Bandura, 2016; Forbes & Stellar, 2022). In contrast, research suggests the existence of a black sheep effect. This effect means that individuals who conform to the norms and values of their ingroup tend to be judged more positively than comparable outgroup members. On the other hand, individuals who deviate from these norms and values tend to be judged more negatively than comparable outgroup members (Marques et al., 1988; Abrams et al., 2013; Bettache et al., 2019). Either way, these psychological findings imply that people are not actually impartial in the way that traditional philosophical theories require.

In addition to the agent, the victim of an immoral act can be either an ingroup member or an outgroup member, and this identity might affect moral judgments of acts that harm that victim. One famous study found that defendants charged with killing white victims were 4.3 times as likely to receive a death sentence than defendants charged with killing black victims, even after taking into account 39 nonracial variables (Baldus et al., 1983; cited in McCleskey v. Kemp 1987). Though capital punishment is a particular case, and sentencing is distinct from moral judgment, this study suggests that people might be less harsh in their moral judgments of lesser wrongs when they see the victim as a member of an outgroup. If so, that would constitute an additional way in which people fail to be impartial in the way required by traditional philosophical theories.

One factor that could affect ingroup versus outgroup moral judgments is cultural collectivism as opposed to individualism. East Asian cultures, such as in traditional parts of China and Korea, are typically seen as more collectivist, whereas Western Cultures, such as in much of the United States and the United Kingdom, are usually classified as more individualist (Hofstede, 1980, 2011; Hofstede et al., 2005). People from collectivist cultures might be expected to show greater differences between their moral judgments of acts that are done by ingroup as opposed to outgroup members or that harm ingroup as opposed to outgroup members.

Of course, individuals within both collectivist and individualist cultures vary in how attached they are to different groups. However, people who feel closer to their families, for example, would on average probably make even more lenient moral judgments of violations by a member of their own family and even harsher moral judgments of harm to a family member (Earp et al., 2021; Lee & Holyoak, 2020; McManus et al., 2020, 2021). In short, psychological closeness can magnify these effects of partiality. In the case of other moral violations and virtues, such as deception and honesty, an interaction between cultural factors and closeness to the agent might occur. Wang et al. (2011) compared how cultural differences impact how individuals reward and punish others. Through three experiments comparing East Asians’ and Americans’ responses to deception and honesty from friends and strangers, they found that East Asians tend to reward honesty more than punish deception when it comes from friends. In contrast, Americans reward more than they punish, regardless of whether it comes from a friend or a stranger.

These effects might also be affected by the nature of the harm to victims. Physical harms, such as those caused by punching a victim, are easier to see than emotional harms, such as those caused by insults or exclusion. Their visibility makes physical harm to outsider victims harder to deny, dismiss, or discount than emotional harm to the same victims. If people feel less confident in assessing the emotions of outsiders, then they might be less inclined to make harsh moral judgments of agents who cause emotional harms to outsiders. As a result, the differences between the moral judgments of insiders and outsiders as agents and victims might be greater for emotional harm than for physical harm.

The goal of the present research is to understand how moral judgments are affected by the identities of agents and victims as insiders or outsiders as well as how these effects are modulated by collectivism, psychological closeness, and the type of harm. We tested several hypotheses that were suggested by the literature:

(H1) *Insider agent effect*: Participants will tend to judge harmful acts by insider agents less harshly than those by outsider agents.

(H2) *Insider victim effect*: Participants will tend to judge harmful acts with insider victims more harshly than those with outsider victims.

(H3) *Collectivism effect*: Participants from more collectivist cultures will show differences in moral judgments compared to participants from more individualist cultures.

(H4) *Closeness effect*: Participants who feel closer to the agent relative to the victim will show larger differences in the insider agent effect (H1), and participants who feel closer to the victim relative to the agent will show larger differences in the insider victim effect (H2).

We will test these hypotheses both for physical and emotional harm.

## Method

### Participants

The sample included 856 participants from Brazil (*Mean age* = 22.6, *SD* = 5.7, range: [18, 62]; Sex: 78.2% females, 21.8% males), 1008 participants from China (*Mean age* = 31.5, *SD* = 7.3, range: [18, 68]; Sex: 52.2% females, 47.8% males), 1776 participants from Korea (*Mean age* = 37.3, *SD* = 11.7, range: [18, 100], 43.9% missing; Sex: 42.4% females, 14.0% males, 43.58% missing), 782 participants from Romania (*Mean age* = 24.0, *SD* = 7.4, range: [18, 84], 0.3% missing; Sex: 61.4% females, 38.6% males,) 995 participants from the United Kingdom (UK; *Mean age* = 37.0, *SD* = 11.9, range: [18, 87], 0.5% missing; Sex: 71.1% females, 28.7% males, 0.20% missing), and 937 participants from the United States (US; *Mean age* = 35.0, *SD* = 11.7, range: [18, 75], 0.1% missing; Sex: 53.9% females, 46.1% males). See Table S1.

Participants were recruited via professional panel providers for the US, Korea, China, and the UK. In Brazil recruitment was done through ads on social media focused on university students. In Romania, a third of participants were recruited online by ads and other means with the remaining two-thirds of participants filling out printed surveys on campuses and other public spaces in Bucharest.

Korean, US, UK, and Chinese participants were given a nominal payment for completing the survey while Brazilian participants received university credit. There was no compensation for Romanian participants.

All participants gave informed consent online and completed the study in their country’s respective native language. This study received full ethical approval from each participating country.

### Vignettes

Twelve vignettes describing moral transgressions were constructed by modifying a subset of the vignettes in Clifford et al. (2015). Half of them involved emotional pain (e.g. “You see a woman making fun of a man for getting dumped by his girlfriend”), while the other half described physical pain (e.g. “You see a woman pouring her hot coffee on a man for insulting her”). Whether emotional or physical pain, each vignette had four versions with the following characteristics:

- OO: the agent and victim were both outgroup members (e.g. stranger hits/insults a stranger),
- OI: the agent was an outgroup member but the victim was an ingroup member (e.g. stranger hits/insults family),
- IO: the agent was an ingroup member but the victim was an outgroup member (e.g. family hits/insults stranger), and
- II: the agent and victim were ingroup members (e.g. family hits/insults family).

See Supplemental Materials for the full set of vignettes.

### Procedure

With an online Qualtrics survey, participants were randomly assigned four vignettes in either emotional or physical pain, one for each of the agent-victim combinations (we will use the term “dyad” to refer to the pairs of agents and victims). After providing demographic information, participants read the four vignettes to which they had been randomly assigned. After each of the four vignettes, participants were asked to make three main judgments:

- I) “How morally wrong is the agent’s behavior?”
- (1 = “not at all wrong” and 100 = “strongly morally wrong”);
- II) “Please circle the picture below which best describes your relationship with the agent described in the scenario.”
- (1 = “Not Close at all” and 5 = “Very Close”);
- III) “Please circle the picture below which best describes your relationship with the victim described in the scenario.”
- (1 = “Not Close at all” and 5 = “Very Close”).

The closeness to agent/victim questions used the Inclusion of Other in Self scale (IOS; Aron et al., 1992). See Supplemental Materials for these measures.

### Analysis

We created a distance composite between agent and victim closeness ratings for each vignette (i.e., Agent distance minus Victim distance) to better capture the relative difference in closeness between both parties involved in the scenarios. Thus, negative values indicated a closer relationship to the victim, while positive values indicated a closer relationship with the agent (a score of zero indicates no difference in closeness to the agent and victim).

Next, we identified outliers, which we defined as any data points that were ± 1.5 *×* IQR from the median. All outliers were then substituted by the inferior or superior value of the 1.5 *×* IQR, as suggested by Field (2013). Any missing values were replaced by the respective variable’s mean with Country and dyad accounted for as factors. All data manipulation and analyses were done in R (R Core Team, 2021).

## Results

### Emotional harm

We fitted a robust linear mixed model fitted by DAStau to predict Moral Wrongness (MW) using a bottom-up approach (i.e., we tested the incremental on our estimates related to each variable). We first built a null model in which we included the participants’ ID number as a random effect (rlmer(MW ~ 1|ID)). Then, we included one variable at a time to our baseline model. The variables were included in the model in the following order: dyad (fixed effects), distance (covariate), country (fixed effects), dyad *×* distance, and political ideology (covariate). We then compare the estimates between each model with the previous one. The inclusion of political ideology did not improve the explanatory power, thus we excluded it (see Tables S2-S7 in the supplemental material). Therefore, the chosen model to predict Moral Wrongness included ‘dyad’, ‘distance’, ‘country’, and ‘dyad *×* distance’, (formula: MW ~ dyad + country + Distance + dyad *×* distance + 1|ID).

The chosen model’s total explanatory power is substantial (conditional *R*^*2*^ = 0.25), and the part related to the fixed effects alone (marginal *R*^*2*^) is 0.09. The model’s intercept, corresponding to dyad = II (ingroup-ingroup), Distance = 0, and Country = Brazil, is at 83.82 (*95% CI* [82.26, 85.39], *t* = 105.11, *p* < 0.001). Table 1 presents the coefficients, confidence intervals, test statistics, and p-values for the comparisons.

**Table 1:** Robust linear mixed model to predict Moral Wrongness of Emotional Harm

Predictors	Estimates	CI	Statistic	*p*
Intercept	83.82	82.26 – 85.39	105.11	< 0.001
Dyad IO	3.45	2.31 – 4.59	5.93	< 0.001
Dyad OI	2.93	1.59 – 4.26	4.31	< 0.001
Dyad OO	-1.88	-3.00 – -0.76	-3.29	0.001
Distance	-3.72	-4.44 – -2.99	-10.09	< 0.001
China	-15.41	-17.31 – -13.51	-15.90	< 0.001
Korea	-4.87	-6.57 – -3.17	-5.62	< 0.001
Romania	-3.20	-5.22 – -1.17	-3.09	0.002
United Kingdom	-10.20	-12.10 – -8.30	-10.53	< 0.001
United States	-15.49	-17.42 – -13.57	-15.79	< 0.001
Dyad IO × Distance	3.54	2.75 – 4.33	8.76	< 0.001
Dyad OI × Distance	1.90	1.07 – 2.74	4.47	< 0.001
Dyad OO × Distance	0.01	-0.93 – 0.95	0.01	0.990
Random effects				
σ^2^	427.76			
τ_00 subNumber_	93.99			
ICC	0.18			
N_subNumber_	3172			
Observations	12,688			
Marginal R^2^ / Conditional R^2^	0.09 / 0.25			

Estimated beta-coefficient, 95% confidence interval, t-statistic, and p-value for the variables in our model

**Fig. 1:**
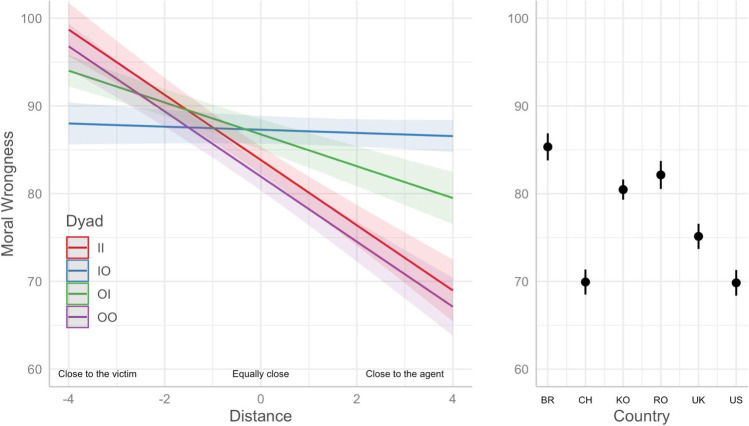
Estimated means for moral wrongness of emotional harm by dyad, distance, and country

After fitting this linear mixed model, pairwise comparisons with Bonferroni adjustment were run on the estimated means. We ran pairwise comparisons for dyad *×* distance considering the values of distance = -4, 0, 4. With this approach, we could verify the differential effects of the dyad as a function of how close participants reported being to the agent and victim (-4 is much closer to the victim, 0 is equally close, and + 4 is much closer to the agent). As shown in Fig. 1; Table 2, the relative distance between agent and victim interacts with the dyad (agent and victim) that made up each moral violation.

**Table 2:** Pairwise comparisons with Bonferroni adjustment on the estimated means

Contrast dyad *×* distance		Estimate	*SE*	z.ratio	*p-value*
II - IO	Closer to victim	10.70	1.70	6.30	< 0.0001
II - OI		4.68	1.52	3.07	0.04
II - OO		1.90	1.80	1.06	1.00
IO - OI		-6.02	1.19	-5.07	< 0.0001
IO - OO		-8.79	1.53	-5.76	< 0.0001
OI - OO		-2.78	1.30	-2.14	0.58
II - IO	Equally close to victim and agent	-3.45	0.58	-5.93	< 0.0001
II - OI		-2.93	0.68	-4.31	< 0.001
II - OO		1.88	0.57	3.29	0.02
IO - OI		0.53	0.69	0.76	1.00
IO - OO		5.33	0.60	8.94	< 0.0001
OI - OO		4.81	0.69	6.97	< 0.0001
II - IO	Closer to agent	-17.60	1.74	-10.13	< 0.0001
II - OI		-10.53	2.10	-5.03	< 0.0001
II - OO		1.85	2.18	0.85	1.00
IO - OI		7.07	1.51	4.67	0.0001
IO - OO		19.46	1.63	11.94	< 0.0001
OI - OO		12.39	1.98	6.27	< 0.000

Pairwise comparisons for dyad *×* distance considering the values of distance = -4 (closer to victim), 0 (equally close to victim and agent), and 4 (closer to agent). Results are averaged across all countries. P-value adjustment: Bonferroni method for 18 tests

The judgment of emotional harm committed by an ingroup against another ingroup or by an outgroup against another outgroup is related to the relative distance from the agent or victim (i.e., greater relative proximity to the victim results in a more severe judgment of the harm and greater proximity to the agent results in a less severe judgment). Compared to dyads composed of an ingroup and an outgroup, dyads from the same group (either ingroup-ingroup or outgroup-outgroup) resulted in both harsher judgments when closer to the victim and milder judgments when closer to the agent. In the case of emotional harm committed by a member of the ingroup against an external member, the greater proximity to the victim resulted in a less severe judgment compared to the judgment made for the other dyads, while the greater proximity to the agent resulted in a more severe judgment compared to the judgment made for the other dyads. That is, an agent from the same group with high proximity is judged more harshly when they emotionally harm someone from the other group compared to when they emotionally harm someone from the same group. When the relative distance is zero (i.e., equally close to the victim and the perpetrator), emotional harm is judged more severely in cases where the dyads are composed of members of different groups (either ingroup-outgroup or outgroup-ingroup).

Figure 1. Estimated means of how emotional harm is judged (moral wrongness) considering the interaction of dyad and distance to the agent and victim and country. The predicted values depicted in the left panel are averaged across all countries. The predicted values depicted in the right panel are averaged across all dyads. Confidence level used: 0.95.

We also ran another pairwise comparison to examine differences between countries. As shown in Fig. 1 (right panel) and Table 3, the estimated values ​​for judgment of emotional harm are higher for Brazil, followed by Romania and Korea, the United Kingdom, the United States, and China. The estimated values ​​for the USA and China were the lowest (less severe judgment of emotional harm) and did not differ from each other.

**Table 3:** Pairwise comparisons with Bonferroni adjustment on the estimated means

Contrast - Countries	Estimate	SE	z.ratio	*p.value*
Brazil - China	15.41	0.97	15.90	< 0.001
Brazil - Korea	4.87	0.87	5.62	< 0.001
Brazil - Romania	3.20	1.03	3.09	0.03
Brazil - United Kingdom	10.20	0.97	10.53	< 0.001
Brazil - United States	15.49	0.98	15.79	< 0.001
China - Korea	-10.54	0.81	-13.00	< 0.001
China - Romania	-12.21	0.99	-12.28	< 0.001
China - United Kingdom	-5.20	0.93	-5.60	< 0.001
China - United States	0.09	0.94	0.09	1.00
Korea - Romania	-1.67	0.89	-1.87	0.91
Korea - United Kingdom	5.33	0.82	6.47	< 0.001
Korea - United States	10.62	0.84	12.68	< 0.001
Romania - United Kingdom	7.01	1.00	7.03	< 0.001
Romania - United States	12.30	1.01	12.20	< 0.001
United Kingdom - United States	5.29	0.94	5.65	< 0.001

* We run pairwise comparisons for the factor country. Results are averaged over levels of dyad. P-value adjustment: Bonferroni method for 15 tests

### Physical harm

We used the same procedures described before regarding the analysis of Emotional Harm. As shown in Tables S8-S13 (supplementary material), the inclusion of political ideology didn’t improve the explanatory power of our model. Thus, the chosen model to predict Moral Wrongness included ‘dyad’, ‘distance’, ‘country’, and ‘dyad *×* distance’ (formula: MW ~ dyad + distance + country + dyad *×* distance + 1|ID). The robust liner model’s total explanatory power is substantial (conditional *R*^*2*^ = 0.408), and the part related to the fixed effects alone (marginal *R*^*2*^) is 0.133. The model’s intercept, corresponding to dyad = II, Distance = 0, and Country = Brazil, is at 91.58 (*95% CI* [90.43, 92.74], *t* = 155.17, *p* < 0.001). Table 4 presents the coefficients, *95% CI*, *t*, and *p*-values for the comparisons.

**Table 4:** Robust linear mixed model to predict moral wrongness of physical harm

Predictors	Estimates	CI	Statistic	*p*
Intercept	91.58	90.43 – 92.74	155.17	< 0.001
Dyad IO	0.08	-0.68 – 0.84	0.20	0.839
Dyad OI	3.90	3.03 – 4.76	8.84	< 0.001
Dyad OO	-2.25	-2.95 – -1.55	-6.29	< 0.001
Distance	-1.87	-2.34 – -1.39	-7.73	< 0.001
China	-15.92	-17.38 – -14.45	-21.31	< 0.001
Korea	-3.53	-4.85 – -2.21	-5.25	< 0.001
Romania	-2.11	-3.66 – -0.56	-2.66	0.008
United Kingdom	-3.97	-5.45 – -2.50	-5.28	< 0.001
United States	-7.51	-9.00 – -6.01	-9.85	< 0.001
Dyad IO × Distance	1.91	1.39 – 2.43	7.20	< 0.001
Dyad OI × Distance	0.65	0.11 – 1.20	2.37	0.018
Dyad OO × Distance	-0.83	-1.45 – -0.21	-2.62	0.009
Random effects				
σ^2^	174.92			
τ_00 subNumber_	81.16			
ICC	0.32			
N_subNumber_	3182			
Observations	12,728			
Marginal R^2^ / Conditional R^2^	0.133 / 0.408			

**Fig. 2:**
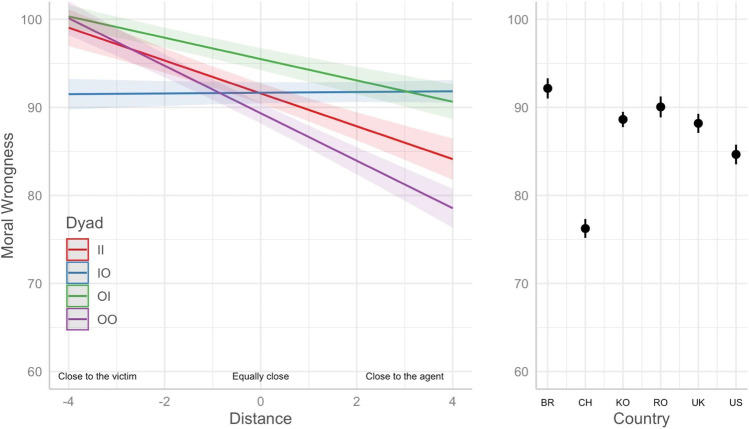
Estimated means for moral wrongness of physical harm by dyad, distance, and country

After that, we ran pairwise comparisons with Bonferroni adjustment on the estimated means. We ran pairwise comparisons for the interaction dyad *×* distance considering the values of the Distance − 4, 0, 4. With this, we could verify the differential effects of the dyad as a function of how relatively close participants reported being to the agent and victim (-4 is close to the victim, 0 is equally close, and + 4 is close to the agent). As shown in Fig. 2 (left panel) and Table 5, the relative distance between agent and victim interacts with the dyad (agent and victim) that made up each moral violation. The moral judgment of physical harm committed by an ingroup against another ingroup (Dyad II), by an outgroup against another outgroup (Dyad OO), and by an outgroup against an ingroup (Dyad OI) are not significantly different from each other but are all significantly harsher than physical harm committed by an ingroup against an outgroup (Dyad IO) when the participant is closer to the victim than to the agent. When relatively closer to the agent, the moral judgment of physical harm committed by an outgroup against another outgroup (Dyad OO) is significantly milder as compared to ingroup against another ingroup (Dyad II) followed by both the moral judgment of physical harm committed by an ingroup against an outgroup (Dyad IO) and by an outgroup against an ingroup (Dyad OI), which are not significantly different from each other. When the relative distance is zero (i.e., equally close to the victim and the perpetrator), physical harm is judged more severely when the agent is from an outgroup and the victim from the ingroup (Dyad OI) compared to all other dyads.

**Table 5:** Pairwise comparisons with Bonferroni adjustment on the estimated means

Contrast dyad *×* distance		estimate	SE	*z.ratio*	*p.value*
II - IO	Closer to victim	7.55	1.13	6.65	*<* 0.0001
II - OI		-1.28	0.99	-1.29	1.0000
II - OO		-1.07	1.21	-0.89	1.0000
IO - OI		-8.83	0.81	-10.90	*<* 0.0001
IO - OO		-8.62	1.06	-8.13	*<* 0.0001
OI - OO		0.21	0.89	0.23	1.0000
II - IO	Equally close to victim and agent	-0.08	0.39	-0.20	1.0000
II - OI		-3.90	0.44	-8.84	*<* 0.0001
II - OO		2.25	0.36	6.29	*<* 0.0001
IO - OI		-3.82	0.46	-8.28	*<* 0.0001
IO - OO		2.33	0.39	5.95	< 0.001
OI - OO		6.15	0.44	13.87	*<* 0.0001
II - IO	Closer to agent	-7.71	1.12	-6.86	*<* 0.0001
II - OI		-6.52	1.36	-4.80	*<* 0.0001
II - OO		5.58	1.42	3.92	< 0.01
IO - OI		1.19	0.97	1.22	1.0000
IO - OO		13.29	1.06	12.59	*<* 0.0001
OI - OO		12.10	1.30	9.32	*<* 0.0001

* We run pairwise comparisons for the interaction dyad *×* distance considering the values of the Distance − 4 (closer to victim), 0 (equally close to victim and agent), and 4 (closer to agent). Results are averaged across countries. P-value adjustment: Bonferroni method for 18 tests

Figure 2. Estimated means of how emotional harm is judged (moral wrongness) considering the interaction of dyad and relative distance to the agent and victim and country. The predicted values depicted in the left panel are averaged across countries. The predicted values depicted in the right panel are averaged over the level of dyad. Confidence level used: 0.95.

We also ran another pairwise comparison to verify differences between countries. As shown in Fig. 2 (right panel) and Table 6, the estimated values ​​for judgment of emotional harm are higher for Brazil, followed by Romania, Korea, the United Kingdom, the United States, and China.

**Table 6:** Pairwise comparisons with Bonferroni adjustment on the estimated means

Contrast - Countries	estimate	SE	*z.ratio*	*p-value*
Brazil - China	15.92	0.75	21.31	*<* 0.0001
Brazil - Korea	3.53	0.67	5.25	*<* 0.0001
Brazil - Romania	2.11	0.79	2.66	0.12
Brazil - United Kingdom	3.97	0.75	5.28	*<* 0.0001
Brazil - United States	7.51	0.76	9.85	*<* 0.0001
China - Korea	-12.39	0.64	-19.32	*<* 0.0001
China - Romania	-13.81	0.77	-17.99	*<* 0.0001
China - United Kingdom	-11.94	0.73	-16.46	*<* 0.0001
China - United States	-8.41	0.74	-11.43	*<* 0.0001
Korea - Romania	-1.42	0.69	-2.05	0.61
Korea - United Kingdom	0.44	0.65	0.68	1.0000
Korea - United States	3.98	0.66	6.00	*<* 0.0001
Romania - United Kingdom	1.87	0.77	2.42	0.24
Romania - United States	5.40	0.78	6.91	*<* 0.0001
United Kingdom - United States	3.53	0.74	4.79	*<* 0.0001

* We run pairwise comparisons for the factor country. Results are averaged over levels of dyad. *P*-value adjustment: Bonferroni method for 15 tests

## Discussion

### General

Our results provide evidence that the insider versus outsider status of agents and victims does affect moral judgments. This asymmetry in moral judgments may have large implications for social interactions. Recent research suggests morality is the primary dimension in which impressions are formed for individuals and groups (Brambilla & Leach, 2014; Brambilla et al., 2011). However, the interactions of these identities with collectivism, psychological closeness, and the type of harm are more complex than traditionally thought. Past research has tended to propose a simplistic general ingroup preference (Bandura, 2016) or a black sheep effect (Marques et al., 1988; Abrams et al., 2013; Bettache et al., 2019), but our findings suggest a more nuanced picture.

This study joins other recent work in challenging the ideal of impartiality—a cornerstone of philosophical moral theories (O’Neill, 2013; Korsgaard, 1998; Wood, 2007; Singer, 2016). One such example is Bloom (2016) which argues children’s impartiality is often compromised by group affiliations. Future studies should aim to incorporate these variables to improve our understanding of the intricacies of our moral evaluations and how they deviate from this philosophical ideal.

### Effects of agent and victim identity

Investigating agent and victim’s identities, our study found robust support for both the insider agent effect (H1) and insider victim effect (H2) such that moral violations committed by outsider agents are generally considered more morally wrong and moral violations committed against an insider victim generally were seen as more morally wrong. These effects were observed consistently across all countries. These findings align with recent studies that report an enhanced ingroup-outgroup bias in children when they hear of a moral transgression being committed by an outsider (Glidden, 2023). Thus, these effects seem to be prevalent not just across national borders but also perhaps across the entire life span.

It’s noteworthy that the insider victim effect intensifies when physical harm is inflicted by an outsider. When comparing the severity of judgment for an ingroup agent causing harm to an outgroup victim (Dyad IO) to the scenario where both agent and victim are insiders (Dyad II), we observed no difference. However, the scenario involving an outsider agent inflicting physical harm to an insider victim (Dyad OI) elicited more severe judgments, while an outgroup agent causing harm to an outgroup victim (Dyad OO) led to less severe judgments. This suggests the victim’s identity gains significance when physical harm is perpetrated by an outsider.

Interestingly, for emotional harm, when both the agent and victim were outgroup members (Dyad OO), the severity of judgment was lesser than when both agent and victim were insiders (Dyad II). Here, the insider victim effect (H2) aligns with our findings of more severe judgments for Dyad II than for Dyad OO. However, the insider agent effect (H1) would suggest less severe judgments for Dyad II than for Dyad OO, which contradicts our findings. This discrepancy may be explained by the insider victim effect (H2) overriding the insider agent effect (H1), suggesting future studies should consider varying both agent and victim identities to capture the full extent of the victim’s identity influence.

A study by Sacchi et al. (2021) highlights the increased self-perception when individuals encounter immoral behavior by outgroup members. Participants exhibited an enhanced self-view, particularly those with strong group affiliations. This suggests that perceiving an outsider’s moral violations as worse than those committed by an insider might contribute to perceived moral superiority and improved self-perceptions. Brambilla and Sacchi (2023) concur, emphasizing the need for future research to examine variations not only in agent identities but also in victim identities.

### Effects of collectivism

The differences in moral judgments between countries of varying levels of collectivism failed to support a collectivism effect (H3). All countries reported less severe moral judgments for both physical and emotional harm compared to Brazil. Romania and Korea were the least different from Brazil, and the US and China were the most different from Brazil in their moral judgments of physical and emotional harm. The UK resembled Romania and Korea for physical harm but the US and China for emotional harm.

This result is inconsistent with Jami and Walker’s (2022) findings, where individuals from collectivist societies displayed higher empathy towards in-group members than those from individualistic societies. Collectivism did not appear to directly influence the pattern of moral judgments in this study. For instance, while Brazil and Romania are close in their level of collectivism (39 and 32, respectively), Korea, despite being highly collectivist (18), reported moral judgments similar to Romania. One would expect China, with a collectivism score of 20, to align with Korea. Instead, it was more similar to the US, which is notably non-collectivist (91). Consequently, our hypothesis that collectivism (H3) would affect judgments of physical or emotional harm was not supported.

### Effects of closeness

Our results affirmed the existence of a ‘closeness effect’ (H4) indicating that participants who perceived greater closeness to either the agents or victims demonstrated increased sensitivity to the insider agent (H1) and insider victim (H2) effects, respectively. This finding aligns with previous research by Cikara et al. (2011) that emphasizes the significant influence of perceived social distance on moral cognition.

In scenarios involving physical harm, we observed that as participants’ perceived closeness to the agent increased, their severity of judgment towards the harmful act diminished, lending further support to the insider agent effect (H1). In parallel, as participants’ perceived closeness to the victim increased, the harmful act was deemed significantly more reprehensible, reinforcing the insider victim effect (H2).

When examining scenarios featuring emotional harm, these effects were amplified. Greater perceived proximity to either the agent or the victim resulted in less severe judgments for the agents and more severe judgments for the victims.

Interestingly, the influence of perceived social distance between the agent and the victim was more pronounced for emotional harm scenarios compared to physical harm. This discrepancy may stem from the fact that physical harm is typically more visibly evident than emotional harm, making it harder for participants to dismiss or deny. Furthermore, participants might have been less certain in assessing the internal emotions of outgroup victims as compared to the visible physical harm, leading to less severe judgments against the agents causing emotional harm.

Adding to this, recent studies provide further insight into the nuanced dynamics of social identity and moral judgment. Čehajić‐Clancy and Bilewicz (2020) observed that perspectives on collective violence can differ significantly between victim and perpetrator groups, further highlighting the influence of victim and agent identity on moral judgment.

### Limitations and future directions

Despite the considerable strengths of our research—such as the large and diverse sample sizes and representation from multiple countries—there are certain limitations that future work should address. First, the compensation for participation varied across countries due to disparate ethical regulations. Participants from Korea, the US, UK, and China received a nominal payment, those from Brazil earned university credits, and Romanian participants were not compensated. This variation might have introduced an unintended bias that could have influenced the outcomes. ​.

Second, future research should incorporate questions to assess participants’ personal collectivist or individualist tendencies, as recent literature challenges oversimplified interpretations of collectivism and individualism based solely on culture (Zhang & Han, 2023; Lomas et al., 2023; Santos et al., 2017). These studies highlight the need to analyze collectivism and individualism attitudes more carefully, considering a broader range of individual features, including moral considerations, to complexify the debate. Our study contributes to this by examining the interplay between psychological closeness, cultural influences, and moral judgments, aiming to enhance our understanding of variations in collectivism and individualism within and across cultures.

Finally, our initial investigation used short vignettes to describe moral violations, which, despite their convenience, might not fully capture the complexity of real-world situations. Future studies should strive to offer richer context around these moral violations and further delineate the identities of outsider agents—perhaps identifying them as part of a specific demographic group. It would also be beneficial to explore the effects of different types of insiders, such as extended family, friends, coworkers, or members of the same religious or political affiliations. By addressing these limitations, future work can further refine our understanding of how identities and cultural factors shape moral judgments.

### Supplementary information

Below is the link to the electronic supplementary material.

ESM 1 (DOCX 37.9 KB)

## Data Availability

All data and code can be found on OSF at: https://osf.io/8xkhs/.
