# Supplementary material for: Does it matter who harmed whom? A cross-cultural study of moral judgments about harm by and to insiders and outsiders
Source: Curr Psychol. 2023 Jul 22;43(9):7997–8007. doi: 10.1007/s12144-023-04986-3 (PMC10965737; doi:10.1007/s12144-023-04986-3)
Supplement: Supplementary file 1 — (DOCX 37.9 KB) [file 12144_2023_4986_MOESM1_ESM.docx]

**Supplemental Materials**

**Experimental designs**

| Eight conditions | | Agent | |
| --- | --- | --- | --- |
|  |  | Ingroup | Outgroup |
| Victim | Ingroup | Emotional pain (EP I-I) | Emotional pain (EP O-I) |
|  |  | Physical pain (PP I-I) | Physical pain (PP O-I) |
|  | Outgroup | Emotional pain (EP I-O) | Emotional pain (EP O-O) |
|  |  | Physical pain (PP I-O) | Physical pain (PP O-O) |

| Mixed design |
| --- |
| --Within-subject for I-O comparison  --Between-subject for emotional-physical pain |
| In this design, there are 12 surveys (6 for an EP, and 6 for PP):  Survey 1 (EP): EP1 OO, EP2 OI, EP3 IO, EP4 II  Survey 2 (EP): EP2 OO, EP3 OI, EP4 IO, EP5 II  Survey 3 (EP): EP3 OO, EP4 OI, EP5 IO, EP6 II  Survey 4 (EP): EP4 OO, EP5 OI, EP6 IO, EP1 II  Survey 5 (EP): EP5 OO, EP6 OI, EP1 IO, EP2 II  Survey 6 (EP): EP6 OO, EP1 OI, EP2 IO, EP3 II  Survey 7 (PP): PP1 OO, PP2 OI, PP3 IO, PP4 II  Survey 8 (PP): PP2 OO, PP3 OI, PP4 IO, PP5 II  Survey 9 (PP): PP3 OO, PP4 OI, PP5 IO, PP6 II  Survey 10 (PP): PP4 OO, PP5 OI, PP6 IO, PP1 II    Survey 11 (PP): PP5 OO, PP6 OI, PP1 IO, PP2 II  Survey 12 (PP): PP6 OO, PP1 OI, PP2 IO, PP3 II |

**Scenarios**

| Moral judgment scenarios for  **Emotional Pain (6 scenarios * 4 conditions)** | |
| --- | --- |
| EP-1 |  |
| OO | You see a woman throwing a gift into the trash right in front of the man who gave her the gift. |
| OI | You see a woman throwing a gift into the trash right in front of your son, who gave her the gift. |
| IO | You see your adult daughter throwing a gift into the trash right in front of the man who gave her the gift. |
| II | You see your adult daughter throwing a gift into the trash right in front of your son, who gave her the gift. |
| EP-2 |  |
| OO | You see a man telling a woman that she looks like a fat pig. |
| OI | You see a man telling your adult daughter that she looks like a fat pig. |
| IO | You see your adult son telling a woman that she looks like a fat pig. |
| II | You see your adult son telling your adult daughter that she looks like a fat pig. |
| EP-3 |  |
| OO | You see a man snickering as he passes by a sick patient because she is thin and frail. |
| OI | You see a man snickering as he passes by your sick mother because she is thin and frail. |
| IO | You see your grown-up son snickering as he passes by a sick patient because she is thin and frail. |
| II | You see your grown-up son snickering as he passes by your sick mother, because she is thin and frail. |
| EP-4 |  |
| OO | You see a dinner guest loudly telling his host that the dinner she cooked tastes awful. |
| OI | You see a dinner guest loudly telling your mother that the dinner she cooked tastes awful. |
| IO | You see your grown-up son loudly telling his host that the dinner she cooked tastes awful. |
| II | You see your grown-up son loudly telling your mother that the dinner she cooked tastes awful. |
| EP-5 |  |
| OO | You see a woman making fun of a man for getting dumped by his girlfriend. |
| OI | You see a woman making fun of your adult son for getting dumped by his girlfriend. |
| IO | You see your grown-up daughter making fun of a man for getting dumped by his girlfriend. |
| II | You see your grown-up daughter making fun of your adult son for getting dumped by his girlfriend. |
| EP-6 |  |
| OO | You see a woman announcing to a man that he looks like a child when he is dancing. |
| OI | You see a woman announcing to your father that he looks like a child when he is dancing. |
| IO | You see your grown-up daughter announcing to a man that he looks like a child when he is dancing. |
| II | You see your grown-up daughter announcing to your father that he looks like a child when he is dancing. |
|  | |
| Moral Judgment Scenarios for  **Physical Pain** (6 scenarios * 4 conditions) | |
| PP-1 |  |
| OO | You see a woman pouring her hot coffee on a man for insulting her. |
| OI | You see a woman pouring her hot coffee on your son for insulting her. |
| IO | You see your mother pouring her hot coffee on a man for insulting her. |
| II | You see your mother pouring her hot coffee on your son for insulting her. |
| PP-2 |  |
| OO | You see a man pushing a woman down a hill for getting in his way. |
| OI | You see a man pushing your daughter down a hill for getting in his way. |
| IO | You see your grown-up son pushing a woman down a hill for getting in his way. |
| II | You see your grown-up son pushing your daughter down the hill for getting in his way. |
| PP-3 |  |
| OO | You see a speaker throwing a shoe at an audience member who is snoring while the speaker is telling a story. |
| OI | You see a speaker throwing a shoe at your spouse who is snoring while the speaker is telling story. |
| IO | You see your adult son throwing a shoe at an audience member who is snoring while the speaker is telling a story. |
| II | You see your adult son throwing a shoe at your spouse who is snoring while the speaker is telling a story. |
| PP-4 |  |
| OO | You see a woman hitting her neighbor for not paying attention while she is telling him what to do for her. |
| OI | You see a woman hitting your son for not paying attention while she is telling him what to do for her. |
| IO | You see your mother hitting her neighbor for not paying attention while she is telling him what to do for her. |
| II | You see your mother hitting your son for not paying attention while she is telling him what to do for her. |
| PP-5 |  |
| OO | You see a woman slapping a man whom she is arguing with in a parking lot. |
| OI | You see a woman slapping your brother whom she is arguing with in a parking lot. |
| IO | You see your sister slapping a man whom she is arguing with in a parking lot. |
| II | You see your sister slapping your brother whom she is arguing with in a parking lot. |
| PP-6 |  |
| OO | You see a man punching his neighbor for walking on the plants in front of his house. |
| OI | You see a man punching your daughter for walking on the plants in front of his house. |
| IO | You see your son punching his neighbor for walking on the plants in front of his house. |
| II | You see your son punching your daughter for walking on the plants in front of his house. |

TABLE S1: Overview of Participants

| Country | Sex  (female) | Age  (Mean (SD)) | Collectivism  Level (0 = Most Collectivistic, 100 = Most Individualistic) | Sample Size |
| --- | --- | --- | --- | --- |
| United States (US) | 53.90% | 35.00 (11.70) | Not Collectivist (91) | 937 |
| United Kingdom (UK) | 71.10% | 37.00 (11.90) | Not Collectivist (89) | 995 |
| Romania | 61.40% | 24.00 (7.40) | Moderately Collectivist (32) | 782 |
| Brazil | 78.20% | 22.60 (5.70) | Moderately Collectivist (39) | 856 |
| Korea | 42.40% | 37.30 (11.70) | Very Collectivist (18) | 1776 |
| China | 52.20% | 31.50 (7.30) | Very Collectivist (20) | 1008 |
| Total | | | | 6,168 |

Note: 43.58% of the Korean sample is missing sex information and 43.90% of the Korean sample is missing age information.

**Statistical Analysis**

Emotional Violations

In our analysis, we assessed the potential multicollinearity among the predictors for three Linear Mixed Effects Regression (LMER) models: a standard LMER, an LMER with Box-Cox lambda correction, and a robust LMER.

Multicollinearity was evaluated considering the full model without the interaction term. This decision was made to prevent possible inflation of Variance Inflation Factors (VIFs) that could be caused by the interaction term. Upon examination, we found similar VIFs for both the LMER and the LMER with Box-Cox lambda correction, with a 95% confidence interval of 1.48 [1.44, 1.51] for the 'dyad', 1.48 [1.45, 1.52] for the 'distance', and 1.01 [1.00, 1.06] for the 'country'. In the robust LMER model, the VIFs were slightly smaller yet comparable, with a 95% confidence interval of 1.46 [1.43, 1.50] for the 'dyad', 1.47 [1.44, 1.51] for the 'distance', and 1.01 [1.00, 1.06] for the 'country'.

We also investigated the Homogeneity of Variance assumption in these three models using Levene’s test. The results suggest that this assumption was met for both the LMER (F = 0.21, p = .65) and the robust LMER (F = 0.83, p = 0.36). However, for the LMER with Box-Cox lambda correction, the assumption was violated (F = 10.85, p < .001). Given the fulfilment of the homogeneity of variance criteria by the LMER and robust LMER and the presence of considerable right skewness in our data, we decided to proceed with the robust LMER model for subsequent analyses. The robust LMER model is particularly advantageous in this situation because it can handle non-normality in the data more effectively than the standard LMER model.

Adding to the factors that influenced the choice of the robust LMER model, we also considered the direction of effects and estimates obtained from the three models as it can be seen in the TABLES S2, S3, S4. Despite the differences in the specifications of the three models (standard LMER, LMER with Box-Cox lambda correction, and robust LMER), the estimates they produced pointed in the same direction. This consistency across models strengthens our confidence in the robustness of our findings. It suggests that the relationships observed between the predictors ('dyad', 'distance', and 'country') and the outcome ('Moral Wrongness') reflect genuine patterns in the data.

In other words, despite the LMER and the robust LMER having slightly different mean squared errors, and the LMER with Box-Cox lambda correction failing the homogeneity of variance test, all three models consistently indicated the same relationships between the predictors and the outcome variable. This convergence of evidence provides further support for selecting the robust LMER model, which has shown to handle non-normality, exhibits good predictive performance, and demonstrates robustness to multicollinearity.

TABLE S2. LMER

|  |  | | | |
| --- | --- | --- | --- | --- |
| *Predictors* | *Estimates* | *CI* | *Statistic* | *p* |
| (Intercept) | 82.41 | 80.61 – 84.21 | 89.63 | **<0.001** |
| dyad [IO] | 4.17 | 2.93 – 5.40 | 6.60 | **<0.001** |
| dyad [OI] | 3.77 | 2.32 – 5.22 | 5.11 | **<0.001** |
| dyad [OO] | -2.17 | -3.38 – -0.95 | -3.50 | **<0.001** |
| Distance | -4.05 | -4.84 – -3.26 | -10.04 | **<0.001** |
| Country [China] | -18.83 | -21.05 – -16.61 | -16.62 | **<0.001** |
| Country [Korea] | -7.66 | -9.65 – -5.67 | -7.56 | **<0.001** |
| Country [Romania] | -4.64 | -7.01 – -2.27 | -3.84 | **<0.001** |
| Country [United Kingdom] | -12.21 | -14.43 – -9.99 | -10.78 | **<0.001** |
| Country [United States] | -18.22 | -20.47 – -15.97 | -15.87 | **<0.001** |
| dyad [IO] × Distance | 3.91 | 3.05 – 4.77 | 8.89 | **<0.001** |
| dyad [OI] × Distance | 2.13 | 1.22 – 3.04 | 4.59 | **<0.001** |
| dyad [OO] × Distance | 0.07 | -0.95 – 1.10 | 0.14 | 0.892 |
| **Random Effects** | | | | |
| σ^2^ | 525.81 | | | |
| τ_00_ _subNumber_ | 157.85 | | | |
| ICC | 0.23 | | | |
| N _subNumber_ | 3172 | | | |
| Observations | 12688 | | | |
| Marginal R^2^ / Conditional R^2^ | 0.087 / 0.298 | | | |
| AIC | 118009.147 | | | |

TABLE S3. LMER_BOX-COX_LAMBDA

|  |  | | | |
| --- | --- | --- | --- | --- |
| *Predictors* | *Estimates* | *CI* | *Statistic* | *p* |
| (Intercept) | 744.76 | 724.02 – 765.50 | 70.38 | **<0.001** |
| dyad [IO] | 45.40 | 31.30 – 59.50 | 6.31 | **<0.001** |
| dyad [OI] | 40.68 | 24.19 – 57.16 | 4.84 | **<0.001** |
| dyad [OO] | -27.42 | -41.24 – -13.60 | -3.89 | **<0.001** |
| Distance | -48.46 | -57.48 – -39.45 | -10.54 | **<0.001** |
| Country [China] | -215.77 | -241.39 – -190.16 | -16.51 | **<0.001** |
| Country [Korea] | -85.22 | -108.12 – -62.32 | -7.29 | **<0.001** |
| Country [Romania] | -52.80 | -80.12 – -25.48 | -3.79 | **<0.001** |
| Country [United Kingdom] | -146.01 | -171.61 – -120.41 | -11.18 | **<0.001** |
| Country [United States] | -213.11 | -239.06 – -187.17 | -16.10 | **<0.001** |
| dyad [IO] × Distance | 46.24 | 36.40 – 56.08 | 9.21 | **<0.001** |
| dyad [OI] × Distance | 25.29 | 14.90 – 35.68 | 4.77 | **<0.001** |
| dyad [OO] × Distance | 1.32 | -10.36 – 13.00 | 0.22 | 0.825 |
| **Random Effects** | | | | |
| σ^2^ | 68266.73 | | | |
| τ_00_ _subNumber_ | 21405.42 | | | |
| ICC | 0.24 | | | |
| N _subNumber_ | 3172 | | | |
| Observations | 12688 | | | |
| Marginal R^2^ / Conditional R^2^ | 0.090 / 0.308 | | | |
| AIC | 179764.658 | | | |

TABLE S4. RLMER

|  |  | | | |
| --- | --- | --- | --- | --- |
| *Predictors* | *Estimates* | *CI* | *Statistic* | *p* |
| (Intercept) | 83.82 | 82.26 – 85.39 | 105.11 | **<0.001** |
| dyad [IO] | 3.45 | 2.31 – 4.59 | 5.93 | **<0.001** |
| dyad [OI] | 2.93 | 1.59 – 4.26 | 4.31 | **<0.001** |
| dyad [OO] | -1.88 | -3.00 – -0.76 | -3.29 | **0.001** |
| Distance | -3.72 | -4.44 – -2.99 | -10.09 | **<0.001** |
| Country [China] | -15.41 | -17.31 – -13.51 | -15.90 | **<0.001** |
| Country [Korea] | -4.87 | -6.57 – -3.17 | -5.62 | **<0.001** |
| Country [Romania] | -3.20 | -5.22 – -1.17 | -3.09 | **0.002** |
| Country [United Kingdom] | -10.20 | -12.10 – -8.30 | -10.53 | **<0.001** |
| Country [United States] | -15.49 | -17.42 – -13.57 | -15.79 | **<0.001** |
| dyad [IO] × Distance | 3.54 | 2.75 – 4.33 | 8.76 | **<0.001** |
| dyad [OI] × Distance | 1.90 | 1.07 – 2.74 | 4.47 | **<0.001** |
| dyad [OO] × Distance | 0.01 | -0.93 – 0.95 | 0.01 | 0.990 |
| **Random Effects** | | | | |
| σ^2^ | 427.76 | | | |
| τ_00_ _subNumber_ | 93.99 | | | |
| ICC | 0.18 | | | |
| N _subNumber_ | 3172 | | | |
| Observations | 12688 | | | |
| Marginal R^2^ / Conditional R^2^ | 0.088 / 0.252 | | | |

We also tested the inclusion of the variable Politics in our models. Tables S5, S6, and S7 present the estimates after including the variable Politics in the models described in tables S2, S3, and S4, respectively. The inclusion of political ideology did not improve the explanatory power. Therefore, the chosen model to predict Moral Wrongness included ‘dyad’, ‘distance’, ‘country’, and ‘dyad *×* distance’, (formula: MW ~ dyad + country + Distance + dyad *×*distance + 1|ID).

TABLE S5. LMER including the variable Politics

| *Predictors* | *Estimates* | *CI* | *Statistic* | *p* |
| --- | --- | --- | --- | --- |
| (Intercept) | 82.19 | 79.47 – 84.91 | 59.18 | **<0.001** |
| dyad [IO] | 4.17 | 2.93 – 5.40 | 6.60 | **<0.001** |
| dyad [OI] | 3.77 | 2.32 – 5.22 | 5.11 | **<0.001** |
| dyad [OO] | -2.17 | -3.38 – -0.95 | -3.50 | **<0.001** |
| Distance | -4.05 | -4.84 – -3.26 | -10.04 | **<0.001** |
| Country [China] | -18.86 | -21.10 – -16.62 | -16.53 | **<0.001** |
| Country [Korea] | -7.67 | -9.66 – -5.68 | -7.56 | **<0.001** |
| Country [Romania] | -4.69 | -7.10 – -2.28 | -3.81 | **<0.001** |
| Country [United Kingdom] | -12.21 | -14.43 – -9.99 | -10.77 | **<0.001** |
| Country [United States] | -18.21 | -20.46 – -15.96 | -15.87 | **<0.001** |
| Politics | 0.06 | -0.51 – 0.64 | 0.21 | 0.833 |
| dyad [IO] × Distance | 3.91 | 3.05 – 4.77 | 8.89 | **<0.001** |
| dyad [OI] × Distance | 2.13 | 1.22 – 3.04 | 4.59 | **<0.001** |
| dyad [OO] × Distance | 0.07 | -0.95 – 1.10 | 0.14 | 0.891 |
| **Random Effects** | | | | |
| σ^2^ | 525.81 | | | |
| τ_00_ _subNumber_ | 157.94 | | | |
| ICC | 0.23 | | | |
| N _subNumber_ | 3172 | | | |
| Observations | 12688 | | | |
| Marginal R^2^ / Conditional R^2^ | 0.087 / 0.298 | | | |
| AIC | 118011.714 | | | |

TABLE S6. LMER_BOX-COX_LAMBDA including the variable Politics

| *Predictors* | *Estimates* | *CI* | *Statistic* | *p* |
| --- | --- | --- | --- | --- |
| (Intercept) | 741.12 | 709.76 – 772.49 | 46.32 | **<0.001** |
| dyad [IO] | 45.40 | 31.30 – 59.50 | 6.31 | **<0.001** |
| dyad [OI] | 40.66 | 24.18 – 57.15 | 4.84 | **<0.001** |
| dyad [OO] | -27.42 | -41.24 – -13.60 | -3.89 | **<0.001** |
| Distance | -48.46 | -57.47 – -39.44 | -10.54 | **<0.001** |
| Country [China] | -216.23 | -242.01 – -190.44 | -16.44 | **<0.001** |
| Country [Korea] | -85.40 | -108.33 – -62.47 | -7.30 | **<0.001** |
| Country [Romania] | -53.58 | -81.37 – -25.79 | -3.78 | **<0.001** |
| Country [United Kingdom] | -145.98 | -171.59 – -120.37 | -11.17 | **<0.001** |
| Country [United States] | -213.08 | -239.03 – -187.13 | -16.10 | **<0.001** |
| Politics | 1.03 | -5.62 – 7.67 | 0.30 | 0.762 |
| dyad [IO] × Distance | 46.24 | 36.40 – 56.08 | 9.21 | **<0.001** |
| dyad [OI] × Distance | 25.28 | 14.89 – 35.67 | 4.77 | **<0.001** |
| dyad [OO] × Distance | 1.32 | -10.36 – 13.01 | 0.22 | 0.824 |
| **Random Effects** | | | | |
| σ^2^ | 68266.78 | | | |
| τ_00_ _subNumber_ | 21416.37 | | | |
| ICC | 0.24 | | | |
| N _subNumber_ | 3172 | | | |
| Observations | 12688 | | | |
| Marginal R^2^ / Conditional R^2^ | 0.090 / 0.308 | | | |
| AIC | 179762.287 | | | |

TABLE S7. RLMER including the variable Politics

| *Predictors* | *Estimates* | *CI* | *Statistic* | *p* |
| --- | --- | --- | --- | --- |
| (Intercept) | 83.47 | 81.13 – 85.81 | 69.88 | **<0.001** |
| dyad [IO] | 3.45 | 2.31 – 4.59 | 5.93 | **<0.001** |
| dyad [OI] | 2.93 | 1.59 – 4.26 | 4.30 | **<0.001** |
| dyad [OO] | -1.88 | -3.00 – -0.76 | -3.29 | **0.001** |
| Distance | -3.72 | -4.44 – -2.99 | -10.08 | **<0.001** |
| Country [China] | -15.45 | -17.36 – -13.54 | -15.84 | **<0.001** |
| Country [Korea] | -4.89 | -6.59 – -3.19 | -5.63 | **<0.001** |
| Country [Romania] | -3.27 | -5.33 – -1.21 | -3.11 | **0.002** |
| Country [United Kingdom] | -10.20 | -12.10 – -8.30 | -10.53 | **<0.001** |
| Country [United States] | -15.49 | -17.41 – -13.57 | -15.79 | **<0.001** |
| Politics | 0.10 | -0.39 – 0.59 | 0.40 | 0.690 |
| dyad [IO] × Distance | 3.54 | 2.75 – 4.33 | 8.76 | **<0.001** |
| dyad [OI] × Distance | 1.90 | 1.07 – 2.74 | 4.47 | **<0.001** |
| dyad [OO] × Distance | 0.01 | -0.93 – 0.95 | 0.01 | 0.989 |
| **Random Effects** | | | | |
| σ^2^ | 427.80 | | | |
| τ_00_ _subNumber_ | 93.94 | | | |
| ICC | 0.18 | | | |
| N _subNumber_ | 3172 | | | |
| Observations | 12688 | | | |
| Marginal R^2^ / Conditional R^2^ | 0.088 / 0.252 | | | |

Physical violations

In our analysis, we assessed the potential multicollinearity among the predictors for three Linear Mixed Effects Regression (LMER) models: a standard LMER, an LMER with Box-Cox lambda correction, and a robust LMER. Multicollinearity was evaluated considering the full model without the interaction term. This decision was made to prevent possible inflation of Variance Inflation Factors (VIFs) that could be caused by the interaction term. Upon examination, we found similar VIFs for the LMER, the LMER with Box-Cox lambda correction and the rlmer: 1.62 [1.58, 1.66] for the 'dyad', 1.63 [1.59, 1.67] for the 'distance', and 1.01 [1.00, 1.08] for the 'country'.

We also investigated the Homogeneity of Variance assumption in these three models using Levene’s test. The results suggest that this assumption wasn’t met for the LMER (F = 13.20, p < 001), the LMER with Box-Cox lambda correction (F = 5.09, p = .02).

and the robust LMER (F = 5.29, p = 0.02). Considering However, for the LMER with Box-Cox lambda correction, the assumption was violated (F = 10.85, p < .001). Given the non-fulfilment of the homogeneity of variance criteria by the LMER and LMER with Box-Cox lambda correction and and the presence of considerable right skewness in our data, we decided to proceed with the robust LMER model for subsequent analyses. The robust LMER model is particularly advantageous in this situation because it can handle non-normality in the data more effectively than the standard LMER model.

Adding to the factors that influenced the choice of the robust LMER model, we also considered the direction of effects and estimates obtained from the three models as it can be seen in the TABLES S8, S9, and S10. Despite the differences in the specifications of the three models (standard LMER, LMER with Box-Cox lambda correction, and robust LMER), the estimates they produced pointed in the same direction. This consistency across models strengthens our confidence in the robustness of our findings. It suggests that the relationships observed between the predictors ('dyad', 'distance', and 'country') and the outcome ('Moral Wrongness') reflect genuine patterns in the data.

In other words, despite the LMER and the robust LMER having slightly different mean squared errors, and the LMER with Box-Cox lambda correction failing the homogeneity of variance test, all three models consistently indicated the same relationships between the predictors and the outcome variable. This convergence of evidence provides further support for selecting the robust LMER model, which has shown to handle non-normality, exhibits good predictive performance, and demonstrates robustness to multicollinearity.

TABLE S8. LMER

|  |  | | | |
| --- | --- | --- | --- | --- |
| *Predictors* | *Estimates* | *CI* | *Statistic* | *p* |
| (Intercept) | 89.99 | 88.59 – 91.39 | 125.72 | **<0.001** |
| dyad [IO] | -0.12 | -1.08 – 0.84 | -0.24 | 0.807 |
| dyad [OI] | 5.91 | 4.83 – 6.99 | 10.70 | **<0.001** |
| dyad [OO] | -2.16 | -3.04 – -1.28 | -4.82 | **<0.001** |
| Distance | -2.28 | -2.87 – -1.68 | -7.55 | **<0.001** |
| Country [China] | -19.19 | -20.95 – -17.42 | -21.31 | **<0.001** |
| Country [Korea] | -5.96 | -7.55 – -4.37 | -7.35 | **<0.001** |
| Country [Romania] | -3.92 | -5.79 – -2.04 | -4.10 | **<0.001** |
| Country [United Kingdom] | -5.16 | -6.94 – -3.38 | -5.68 | **<0.001** |
| Country [United States] | -10.24 | -12.04 – -8.44 | -11.14 | **<0.001** |
| dyad [IO] × Distance | 2.32 | 1.67 – 2.97 | 7.01 | **<0.001** |
| dyad [OI] × Distance | 0.89 | 0.21 – 1.56 | 2.57 | **0.010** |
| dyad [OO] × Distance | -0.67 | -1.45 – 0.11 | -1.69 | 0.092 |
| **Random Effects** | | | | |
| σ^2^ | 289.27 | | | |
| τ_00_ _subNumber_ | 118.72 | | | |
| ICC | 0.29 | | | |
| N _subNumber_ | 3182 | | | |
| Observations | 12728 | | | |
| Marginal R^2^ / Conditional R^2^ | 0.122 / 0.377 | | | |
| AIC | 111362.861 | | | |

TABLE S9. LMER_BOX-COX_LAMBDA

|  |  | | | |
| --- | --- | --- | --- | --- |
| *Predictors* | *Estimates* | *CI* | *Statistic* | *p* |
| (Intercept) | 4193.96 | 4093.77 – 4294.14 | 82.05 | **<0.001** |
| dyad [IO] | -4.41 | -70.65 – 61.83 | -0.13 | 0.896 |
| dyad [OI] | 369.88 | 294.88 – 444.88 | 9.67 | **<0.001** |
| dyad [OO] | -167.75 | -228.69 – -106.80 | -5.40 | **<0.001** |
| Distance | -169.18 | -210.26 – -128.10 | -8.07 | **<0.001** |
| Country [China] | -1317.34 | -1444.05 – -1190.63 | -20.38 | **<0.001** |
| Country [Korea] | -417.36 | -531.54 – -303.19 | -7.17 | **<0.001** |
| Country [Romania] | -257.71 | -392.20 – -123.23 | -3.76 | **<0.001** |
| Country [United Kingdom] | -395.94 | -523.64 – -268.23 | -6.08 | **<0.001** |
| Country [United States] | -744.95 | -874.29 – -615.60 | -11.29 | **<0.001** |
| dyad [IO] × Distance | 172.23 | 127.16 – 217.30 | 7.49 | **<0.001** |
| dyad [OI] × Distance | 54.15 | 7.21 – 101.09 | 2.26 | **0.024** |
| dyad [OO] × Distance | -46.84 | -100.88 – 7.19 | -1.70 | 0.089 |
| **Random Effects** | | | | |
| σ^2^ | 1385875.87 | | | |
| τ_00_ _subNumber_ | 638050.43 | | | |
| ICC | 0.32 | | | |
| N _subNumber_ | 3182 | | | |
| Observations | 12728 | | | |
| Marginal R^2^ / Conditional R^2^ | 0.120 / 0.397 | | | |
| AIC | 219347.512 | | | |

TABLE S10. RLMER

|  |  | | | |
| --- | --- | --- | --- | --- |
| *Predictors* | *Estimates* | *CI* | *Statistic* | *p* |
| Intercept | 91.58 | 90.43 – 92.74 | 155.17 | **<0.001** |
| Dyad IO | 0.08 | -0.68 – 0.84 | 0.20 | 0.839 |
| Dyad OI | 3.90 | 3.03 – 4.76 | 8.84 | **<0.001** |
| Dyad OO | -2.25 | -2.95 – -1.55 | -6.29 | **<0.001** |
| Distance | -1.87 | -2.34 – -1.39 | -7.73 | **<0.001** |
| China | -15.92 | -17.38 – -14.45 | -21.31 | **<0.001** |
| Korea | -3.53 | -4.85 – -2.21 | -5.25 | **<0.001** |
| Romania | -2.11 | -3.66 – -0.56 | -2.66 | **0.008** |
| United Kingdom | -3.97 | -5.45 – -2.50 | -5.28 | **<0.001** |
| United States | -7.51 | -9.00 – -6.01 | -9.85 | **<0.001** |
| Dyad IO × Distance | 1.91 | 1.39 – 2.43 | 7.20 | **<0.001** |
| Dyad OI × Distance | 0.65 | 0.11 – 1.20 | 2.37 | **0.018** |
| Dyad OO × Distance | -0.83 | -1.45 – -0.21 | -2.62 | **0.009** |
| **Random Effects** | | | | |
| σ^2^ | 174.92 | | | |
| τ_00_ _subNumber_ | 81.16 | | | |
| ICC | 0.32 | | | |
| N _subNumber_ | 3182 | | | |
| Observations | 12728 | | | |
| Marginal R^2^ / Conditional R^2^ | 0.133 / 0.408 | | | |

We also tested the inclusion of the variable Politics in our models. Tables S11, S12, and S13 present the estimates after including the variable Politics in the models described in tables S8, S9, and S10, respectively. The inclusion of political ideology did not improve the explanatory power. Therefore, the chosen model to predict Moral Wrongness included ‘dyad’, ‘distance’, ‘country’, and ‘dyad *×* distance’, (formula: MW ~ dyad + country + Distance + dyad *×*distance + 1|ID).

TABLE S11. LMER including the variable Politics

| *Predictors* | *Estimates* | *CI* | *Statistic* | *p* |
| --- | --- | --- | --- | --- |
| (Intercept) | 89.84 | 87.69 – 91.99 | 81.84 | **<0.001** |
| dyad [IO] | -0.12 | -1.07 – 0.84 | -0.24 | 0.807 |
| dyad [OI] | 5.91 | 4.83 – 6.99 | 10.70 | **<0.001** |
| dyad [OO] | -2.16 | -3.04 – -1.28 | -4.82 | **<0.001** |
| Distance | -2.28 | -2.87 – -1.69 | -7.55 | **<0.001** |
| Country [China] | -19.21 | -20.99 – -17.43 | -21.17 | **<0.001** |
| Country [Korea] | -5.97 | -7.56 – -4.38 | -7.35 | **<0.001** |
| Country [Romania] | -3.95 | -5.85 – -2.05 | -4.07 | **<0.001** |
| Country [United Kingdom] | -5.15 | -6.93 – -3.37 | -5.67 | **<0.001** |
| Country [United States] | -10.23 | -12.04 – -8.43 | -11.12 | **<0.001** |
| Politics | 0.04 | -0.42 – 0.50 | 0.18 | 0.854 |
| dyad [IO] × Distance | 2.32 | 1.67 – 2.97 | 7.01 | **<0.001** |
| dyad [OI] × Distance | 0.89 | 0.21 – 1.56 | 2.58 | **0.010** |
| dyad [OO] × Distance | -0.67 | -1.45 – 0.11 | -1.68 | 0.093 |
| **Random Effects** | | | | |
| σ^2^ | 289.27 | | | |
| τ_00_ _subNumber_ | 118.78 | | | |
| ICC | 0.29 | | | |
| N _subNumber_ | 3182 | | | |
| Observations | 12728 | | | |
| Marginal R^2^ / Conditional R^2^ | 0.122 / 0.377 | | | |
| AIC | 111365.894 | | | |

TABLE S12. LMER_BOX-COX_LAMBDA including the variable Politics

| *Predictors* | *Estimates* | *CI* | *Statistic* | *p* |
| --- | --- | --- | --- | --- |
| (Intercept) | 4191.79 | 4037.67 – 4345.91 | 53.31 | **<0.001** |
| dyad [IO] | -4.40 | -70.64 – 61.84 | -0.13 | 0.896 |
| dyad [OI] | 369.88 | 294.88 – 444.88 | 9.67 | **<0.001** |
| dyad [OO] | -167.74 | -228.69 – -106.79 | -5.39 | **<0.001** |
| Distance | -169.20 | -210.28 – -128.11 | -8.07 | **<0.001** |
| Country [China] | -1317.63 | -1445.29 – -1189.96 | -20.23 | **<0.001** |
| Country [Korea] | -417.42 | -531.66 – -303.19 | -7.16 | **<0.001** |
| Country [Romania] | -258.16 | -394.82 – -121.50 | -3.70 | **<0.001** |
| Country [United Kingdom] | -395.82 | -523.69 – -267.96 | -6.07 | **<0.001** |
| Country [United States] | -744.85 | -874.32 – -615.38 | -11.28 | **<0.001** |
| Politics | 0.61 | -32.32 – 33.54 | 0.04 | 0.971 |
| dyad [IO] × Distance | 172.24 | 127.17 – 217.31 | 7.49 | **<0.001** |
| dyad [OI] × Distance | 54.16 | 7.22 – 101.10 | 2.26 | **0.024** |
| dyad [OO] × Distance | -46.82 | -100.86 – 7.22 | -1.70 | 0.089 |
| **Random Effects** | | | | |
| σ^2^ | 1385875.48 | | | |
| τ_00_ _subNumber_ | 638361.09 | | | |
| ICC | 0.32 | | | |
| N _subNumber_ | 3182 | | | |
| Observations | 12728 | | | |
| Marginal R^2^ / Conditional R^2^ | 0.120 / 0.397 | | | |
| AIC | 219342.031 | | | |

TABLE S13. RLMER including the variable Politics

| *Predictors* | *Estimates* | *CI* | *Statistic* | *p* |
| --- | --- | --- | --- | --- |
| (Intercept) | 91.65 | 89.87 – 93.43 | 100.92 | **<0.001** |
| dyad [IO] | 0.08 | -0.68 – 0.84 | 0.20 | 0.839 |
| dyad [OI] | 3.90 | 3.03 – 4.76 | 8.84 | **<0.001** |
| dyad [OO] | -2.25 | -2.96 – -1.55 | -6.29 | **<0.001** |
| Distance | -1.87 | -2.34 – -1.39 | -7.73 | **<0.001** |
| Country [China] | -15.91 | -17.38 – -14.43 | -21.14 | **<0.001** |
| Country [Korea] | -3.53 | -4.85 – -2.21 | -5.24 | **<0.001** |
| Country [Romania] | -2.10 | -3.67 – -0.52 | -2.60 | **0.009** |
| Country [United Kingdom] | -3.98 | -5.45 – -2.50 | -5.28 | **<0.001** |
| Country [United States] | -7.51 | -9.01 – -6.01 | -9.84 | **<0.001** |
| Politics | -0.02 | -0.40 – 0.36 | -0.09 | 0.927 |
| dyad [IO] × Distance | 1.91 | 1.39 – 2.43 | 7.20 | **<0.001** |
| dyad [OI] × Distance | 0.65 | 0.11 – 1.20 | 2.37 | **0.018** |
| dyad [OO] × Distance | -0.83 | -1.45 – -0.21 | -2.62 | **0.009** |
| **Random Effects** | | | | |
| σ^2^ | 174.91 | | | |
| τ_00_ _subNumber_ | 81.20 | | | |
| ICC | 0.32 | | | |
| N _subNumber_ | 3182 | | | |
| Observations | 12728 | | | |
| Marginal R^2^ / Conditional R^2^ | 0.133 / 0.408 | | | |
